# Affective profiles in Italian high school students: life satisfaction, psychological well-being, self-esteem, and optimism

**DOI:** 10.3389/fpsyg.2015.01310

**Published:** 2015-09-01

**Authors:** Annamaria Di Fabio, Ornella Bucci

**Affiliations:** Department of Education and Psychology, University of FlorenceFlorence, Italy

**Keywords:** affective profiles, life satisfaction, psychological well-being, self-esteem, optimism

## Abstract

The affective profiles model distinguishes between individuals who are self-fulfilling (high positive affect, low negative affect), high affective (high positive affect, high negative affect), low affective (low positive affect, low negative affect), and self-destructive (low positive affect, high negative affect). The literature shows that the affective profiles model has been used with Swedish people in particular in order to determine differences among profiles in relation to life satisfaction, psychological well-being, self-esteem, and optimism. The present research investigated these differences in Italian high school students. Two studies were conducted: the first with 156 Italian high school students and the second with 148 Italian high school students. The first study analyzed differences among affective profiles with regard to life satisfaction and psychological well-being while the second study analyzed differences among affective profiles with regard to self-esteem and optimism. In the first study, the Positive and Negative Affect Schedule (PANAS), the Satisfaction with Life Scale, and the Meaningful Life Measure were administered to the participants. In the second study, the PANAS, the Self-Esteem Scale, the Life Orientation Test-revised were administered to the participants. The results of the first study showed that, with respect to the other profiles, the self-fulfilling participants had greater life satisfaction and psychological well-being. The results of the second study showed that, with respect to the other profiles, the self-fulfilling participants had higher self-esteem and optimism. These results revealed differences among affective profiles regarding life satisfaction, psychological well-being, self-esteem, and optimism in the Italian context as well thereby offering new possibilities for cross-cultural research and for enhancing self-fulfilling profiles.

## Introduction

Watson et al. (1988) traditional model holds that all emotional experiences can be explained by two affective dimensions: positive affect (PA) and negative affect (NA). These two dimensions are independent, and, accordingly, an increase in PA does not necessarily correspond with a decrease in NA (Watson and Tellegen, 1985; Tellegen et al., 1999). The assessment of affectivity as a continuum does not always enable the accurate detection of the different positive and negative aspects of emotional experience (Schimmack and Diener, 1997). However, the Positive and Negative Affect Schedule (PANAS, Watson et al., 1988), the most commonly used tool to measure affect, regards PA and NA as orthogonal independent factors: high PA against low PA and high NA against low NA (Watson and Tellegen, 1985). Using self-reported affect as measured by the PANAS, Norlander et al. (2002, 2005) and Archer et al. (2007) developed the affective profile model that includes the self-fulfilling profile (high PA, low NA), the high affective profile (high PA, high NA), the low affective profile (low PA, low NA), and the self-destructive profile (low PA, high NA). This model does not treat PA and NA as two distinct dimensions and consequently offers a more specific and detailed description of PA and NA (Garcia, 2012).

Most of the studies on affective profiles have been carried out among Swedes (Archer et al., 2007, 2008; Garcia and Siddiqui, 2009a,b; Garcia et al., 2014) and have shown that self-fulfilling individuals generally believe that they are more energetic and optimistic than individuals with the other three affective profiles (Archer et al., 2007). Individuals with self-fulfilling and high affective profiles perform best during stressful situations and demonstrate a more dynamic lifestyle than low affective and self-destructive individuals (Norlander et al., 2002, 2005). Self-fulfilling individuals report greater life satisfaction and psychological well-being than individuals with the other affective profiles (Garcia and Siddiqui, 2009a,b). They also obtain higher scores in variables relating to agentic values (e.g., autonomy, responsibility, self-acceptance, internal locus of control, and self-control) than the other individuals (Garcia, 2012). The different affective profiles reflect also differences in self-esteem, optimism, and locus of control (Archer et al., 2008). For example, individuals with self-fulfilling profiles reveal high self-esteem, high optimism, and internal locus of control, whereas individuals with self-destructive profiles reveal low self-esteem, low optimism, and external locus of control (Archer et al., 2008).

Similar studies have been conducted with Dutch (Kunst, 2011), American (Schütz et al., 2013), Iranian (Garcia and Moradi, 2013), Indonesian (Adrianson et al., 2013), and Salvadorian (Garcia et al., 2015) participants using different variables in relation to the four affective profiles. Kunst’s (2011) study revealed that the self-destructive and high affective profiles were strongly associated with more severe post-traumatic stress disorder symptoms in Dutch victims of violence. Schütz et al. (2013) found in the case of their US participants that, with respect to the other profiles, the self-fulfilling participants perceived themselves as less depressed, happier, and more satisfied with their lives. Conversely, the self-destructive participants perceived themselves as more depressed, unhappier, and less satisfied with their lives compared to the participants with the other profiles (Schütz et al., 2013). Individuals with self-fulfilling profiles tend to use strategies based on agentic (i.e., instrumental goal pursuit), communal (i.e., social affiliation), and spiritual (i.e., religious) values (Tkach and Lyubomirsky, 2006) in the pursuit of happiness (Schütz et al., 2013). Garcia and Moradi’s (2013) study, which compared the affective profiles of Swedish and Iranian adolescents, revealed that across cultures the self-fulfilling participants reported greater life satisfaction and psychological well-being. In another study, the self-fulfilling Swedish and Indonesian participants (Adrianson et al., 2013) demonstrated greater self-esteem and optimism and less impulsiveness. In Garcia et al.’s (2015) research involving Salvadorian participants, the self-destructive participants reported lower levels of self-directedness than the participants with the other profiles, while cooperativeness was higher only among the self-fulfilling participants. It is essential to continue studying affective profiles in different international contexts to see if the model produces similar results in different populations, to verify the validity of the model across international contexts, and to promote cross-cultural research (Garcia and Moradi, 2013; Garcia et al., 2015). The literature indicates that the affective profiles model has been used to identify differences among profiles regarding life satisfaction, psychological well-being, self-esteem, and optimism in different international contexts (Archer et al., 2008; Garcia and Siddiqui, 2009a,b; Adrianson et al., 2013; Garcia and Moradi, 2013; Schütz et al., 2013) but that no such studies have up till now been done with Italian participants. The two studies discussed in this article are the first to have analyzed differences among affective profiles in relation to life satisfaction, psychological well-being, self-esteem, and optimism in the Italian context. The research subjects were high school students in their final year of school. These students were chosen because they were at a critical stage of their lives when they had to make important decisions regarding their careers and future lives with crucial implications for their life satisfaction and psychological well-being on the one hand and their self-esteem and optimism on the other. Two studies were carried out: the first analyzed differences among affective profiles with regard to life satisfaction and psychological well-being, and the second analyzed differences among affective profiles with regard to self-esteem and optimism.

## First Study

### Aim and Hypotheses

In terms of the above theoretical framework, the first study investigated differences among affective profiles in relation to life satisfaction and psychological well-being in Italian high school students in line with studies conducted earlier with Swedish participants (Garcia and Siddiqui, 2009a,b; Garcia and Moradi, 2013), Iranian participants (Garcia and Moradi, 2013), and American participants (Schütz et al., 2013). The following hypotheses were formulated.

H1: Self-fulfilling individuals have greater life satisfaction than individuals with the other profiles.
H2: Self-fulfilling individuals have greater psychological well-being than individuals with the other profiles.

### Materials and Methods

#### Participants

One hundred and fifty-six students in their final year of high school in the Tuscan school system participated in the study. All high school students in their final year in the school system were invited to take part in the study. Regarding gender, 64 (41.03%) of the participants were males and 92 (58.97%) were females. The age of the participants ranged from 18 to 20 years (*M* = 19.76, *SD* = 0.49).

#### Measures

##### Positive and Negative Affect Schedule

The Italian version of the Positive Affect Scale of the PANAS by Terracciano et al. (2003; Watson et al., 1988) was used to evaluate PA and NA. The PANAS consists of a list of 20 adjectives of which 10 refer to PA (e.g., enthusiastic, interested, and determined) and 10 to NA (e.g., afraid, upset, and distressed). The participants were asked to indicate the intensity they generally felt on a Likert scale ranging from 1 = *Very slightly or not* at all to 5 = *Extremely*. The Cronbach’s alpha coefficients were 0.72 for PA and 0.83 for NA.

##### Satisfaction with Life Scale (SWLS)

The Italian version by Di Fabio and Busoni (2009) of the Satisfaction with Life Scale (SWLS, Diener et al., 1985) was used to evaluate life satisfaction. The questionnaire has five items on a 7-point Likert scale ranging from 1 = *Strongly disagree* to 7 = *Strongly agree*. The confirmatory factor analysis carried out on the Italian version of the SWLS revealed a one-dimensional factorial structure for the original version of the scale. The Cronbach’s alpha coefficient was 0.88.

##### Meaningful Life Measure (MLM)

The Italian version by Di Fabio (2014b) of the Meaningful Life Measure (MLM, Morgan and Farsides, 2009) was used to evaluate life meaning. The questionnaire consists of 23 items on a 7-point Likert scale ranging from 1 = *Strongly disagree* to 7 = *Strongly agree*. The MLM identifies the five dimensions of life meaningfulness: exciting life, accomplished life, principled life, purposeful life, and valued life. The Cronbach’s alpha coefficient for the total score used in the present study was 0.81.

#### Procedure and Data Analysis

The instruments were administered collectively in the classroom by trained psychologists. The order of administration was counterbalanced to control the effects of presentation.

The instruments were administered at a time decided on by the school and in accordance with the requirements of privacy and informed consent laid down by Italian law (Law Decree DL-196/2003). With regard to ethical standards for research, the study adhered to the latest version of the Declaration of Helsinki revised in Fortaleza (World Medical Association [WMA], 2013).

A median split categorizing the participants’ PA and NA scores into high and low was done in order to divide the participants into different affective profiles. Following this, different profile combinations were created using the high/low PA and NA categories: self-fulfilling (high PA and low NA), low affective (low PA and low NA), high affective (high PA and high NA), and self-destructive (low PA and high NA). Archer and his colleagues (Norlander et al., 2002, 2005) devised this procedure to obtain different affective profiles. A multivariate analysis of variance (MANOVA) was carried out to analyze the differences in life satisfaction and psychological well-being among Italian high school students with different affective profiles. Bonferroni’s *post hoc* test was used to investigate differences in the different profiles in the dependent variables.

### Results

The MANOVA results highlighted the significant effect of the affective profiles on life satisfaction and psychological well-being in terms of life meaning: *F*(3,152) = 8.58, *p* < 0.001, Wilks’ Lambda = 0.73, η^2^ = 0.15.

Specifically the following significant effect of the affective profiles on life satisfaction emerged: *F*(3,152) = 8.94, *p* < 0.001, η^2^ = 0.15. Bonferroni’s *post hoc* test revealed that the high school students with self-fulfilling profiles scored higher (*p* < 0.001) in life satisfaction (*M* = 23.98, *SD* = 5.88) than the high school students with high affective (*M* = 20.19, *SD* = 6.34), low affective (*M* = 20.15, *SD* = 5.04), and self-destructive profiles (*M* = 17.81, *SD* = 5.18; **Table 1**).

**Table 1:** Means differences in life satisfaction and well-being in affective profiles.

Self-fulfilling (*n* = 48)		High affective (*n* = 31)		Low positive (*n* = 40)		Self-destructive (*n* = 37)		*F*(3,152)	η^2^
*M*	*SD*	*M*	*SD*	*M*	*SD*	*M*	*SD*		
23.98^bcd^	5.88	20.19^ad^	6.34	20.15^ad^	5.04	17.81^abc^	5.18	8.94^∗∗∗^	0.15
112.38^bcd^	17.51	100.68^abc^	14.08	95.68^abc^	13.42	92.22^abc^	14.21	14.92^∗∗∗^	0.23

*N = 156 ; ^∗∗∗^*p* < 0.001; ^a^Self-fulfilling, ^b^High affective, ^c^Low positive, and ^d^Self-destructive*.

The affective profiles also differed with regard to psychological well-being in terms of life meaning: *F*(3,152) = 14.92, *p* < 0.001, η^2^ = 0.23. Bonferroni’s *post hoc* test revealed that the high school students with self-fulfilling profiles scored higher (*p* < 0.001) in psychological well-being (*M* = 112.38, *SD* = 17.51) than the high school students with high affective (*M* = 100.68, *SD* = 14.08), low affective (*M* = 95.68, *SD* = 13.42), and self-destructive profiles (*M* = 92.22, *SD* = 14.21; **Table 1**).

## Second Study

### Aim and Hypotheses

In terms of the theoretical framework discussed earlier, the second study investigated differences among affective profiles in relation to self-esteem and optimism in Italian high school students in line with earlier studies carried out by Archer et al. (2008) among Swedish participants and by Adrianson et al. (2013) among Swedish as well as Indonesian participants. The following hypotheses were formulated.

H1: Self-fulfilling individuals have higher self-esteem compared to individuals with the other profiles.
H2: Self-fulfilling individuals are more optimistic compared to individuals with the other profiles.

### Materials and Methods

#### Participants

One hundred and forty-eight students in their final year of high school in the Tuscan school system participated in the present study. All high school students in their final year in the school system were invited to take part in the study. Regarding gender, 59 (39.86%) of the participants were males and 89 (60.14%) were females. The age of the participants ranged from 18 to 20 years (*M* = 19.24, *SD* = 0.73).

#### Measures

##### Positive and Negative Affect Schedule

The Italian version by Terracciano et al. (2003) of the Positive Affect Scale of the PANAS (Watson et al., 1988) was used to evaluate PA and NA. The PANAS consists of a list of 20 adjectives of which 10 refer to PA (e.g., enthusiastic, interested, and determined) and 10 refer to NA (e.g., afraid, upset, and distressed). The participants were asked to indicate the intensity they generally felt on a Likert scale ranging from 1 = *Very slightly or not* at all to 5 = *Extremely*. The Cronbach’s alpha coefficients were 0.72 for PA and 0.83 for NA.

##### Self-Esteem Scale (SES)

The Italian version by Prezza et al. (1997) of the Self-Esteem Scale (SES, Rosenberg, 1965) was used to evaluate self-esteem. The scale consists of 10 items on a 4-point Likert scale ranging from 1 = *Strongly disagree* to 4 = *Strongly agree*. The Italian version of the SES has a one-dimensional factorial structure. The Cronbach’s alpha coefficient was 0.84.

##### Life Orientation Test-revised (LOT-r)

The Italian version by Giannini et al. (2008) of the Life Orientation Test-revised (LOT-r, Scheier and Carver, 1985) was used to evaluate optimism. This scale consists of 10 items on a 5-point Likert scale ranging from 0 = *Strongly disagree* to 4 = *Strongly agree*. The Italian version of the LOT-r has a one-dimensional factorial structure. The Cronbach’s alpha coefficient was 0.81.

#### Procedure and Data Analysis

The instruments were administered collectively in the classroom by trained psychologists. The order of administration was counterbalanced to control the effects of presentation.

The instruments were administered at a time decided on by the school and in accordance with the requirements of privacy and informed consent laid down by Italian law (Law Decree DL-196/2003).With regard to the ethical standards for research, the study adhered to the latest version of the Declaration of Helsinki revised in Fortaleza (World Medical Association [WMA], 2013).

A median split categorizing the participants’ PA and NA scores into high and low was done in order to divide the participants into different affective profiles. Following this, different profile combinations were created using the high/low PA and NA categories: self-fulfilling (high PA and low NA), low affective (low PA and low NA), high affective (high PA and high NA), and self-destructive (low PA and high NA). Archer and his colleagues (Norlander et al., 2002, 2005) devised this procedure to obtain different affective profiles. A MANOVA was carried out to analyze the differences in self-esteem and optimism among Italian high school students with different affective profiles. Bonferroni’s *post hoc* test was used to investigate the differences among the different profiles in the dependent variables.

### Results

The MANOVA results highlighted the significant effect of the affective profiles on self-esteem and optimism: *F*(3,144) = 8.79, *p* < 0.01, Wilks’ Lambda = 0.87, η^2^ = 0.07.

Specifically the following significant effect of the affective profiles on self-esteem emerged: *F*(3,144) = 5.21, *p* < 0.01, η^2^ = 0.09. Bonferroni’s *post hoc* test revealed that the high school students with self-fulfilling profiles scored higher (*p* < 0.001) in self-esteem (*M* = 32.24, *SD* = 4.79) than the high school students with high affective (*M* = 29.29, *SD* = 4.80), low affective (*M* = 29.30, *SD* = 3.41), and self-destructive profiles (*M* = 30.19, *SD* = 4.13; **Table 2**).

**Table 2:** Means differences in self-esteem and optimism in affective profiles.

Self-fulfilling (*n* = 45)		High affective (*n* = 32)		Low positive (*n* = 37)		Self-destructive (*n* = 34)		*F*(3,144)	η^2^
*M*	*SD*	*M*	*SD*	*M*	*SD*	*M*	*SD*		
32.24^bcd^	4.79	29.29^a^	4.80	29.30^a^	3.41	30.19^a^	4.13	5.21^∗∗^	0.09
19.87^bcd^	3.87	17.52^a^	3.82	17.70^a^	3.03	17.76^a^	2.86	4.34^∗∗^	0.08

**N* = 148; ^∗∗^*p* < 0.001; ^a^Self-fulfilling, ^b^High affective, ^c^Low positive, and ^d^Self-destructive*.

The affective profiles differed also with regard to optimism: *F*(3,144) = 4.34, *p* < 0.01, η^2^ = 0.08. Bonferroni’s *post hoc* test revealed that the high school students with self-fulfilling profiles scored higher (*p* < 0.001) in optimism (*M* = 19.87, *SD* = 3.87) than the high school students with high affective (*M* = 17.52, *SD* = 3.82), low affective (*M* = 17.70, *SD* = 3.03), and self-destructive profiles (*M* = 17.76, *SD* = 2.86; **Table 2**).

## Discussion and Conclusion

The literature highlights the need to continue studying affective profiles in different international contexts to see if the affective profiles model produces similar results in different populations (Garcia and Moradi, 2013; Garcia et al., 2015). The two studies discussed in this article were the first to analyze differences among affective profiles in relation to life satisfaction, psychological well-being, self-esteem, and optimism in an Italian context.

The first study examined differences among affective profiles in relation to life satisfaction and psychological well-being in Italian high school students.

The first hypothesis of the first study was confirmed as the participants with self-fulfilling profiles had greater life satisfaction than the participants with the other affective profiles. These results are in line with those of studies carried out with Swedish participants (Garcia and Siddiqui, 2009a,b; Garcia and Moradi, 2013), Iranian participants (Garcia and Moradi, 2013), and American participants (Schütz et al., 2013).

Individuals with more positive emotions with regard to pleasure attainment and fewer negative emotions with regard to pain avoidance are generally more satisfied with their lives. Individuals with a high PA and at the same time a low NA therefore tend to be more satisfied with their lives (Garcia and Siddiqui, 2009a,b; Garcia and Moradi, 2013; Schütz et al., 2013).

The second hypothesis of the first study was also confirmed as the participants with self-fulfilling profiles demonstrated greater psychological well-being than the participants with the other profiles. These results are in line with those of studies carried out with Swedish participants (Garcia and Siddiqui, 2009b; Garcia and Moradi, 2013) and Iranian participants (Garcia and Moradi, 2013). Individuals who experience more positive emotions in terms of high PA and low NA generally have greater psychological well-being thus indicating that individuals who seek pleasure and avoid pain tend to experience greater psychological well-being in terms of life meaning (Morgan and Farsides, 2009).

The second study examined differences among affective profiles in relation to self-esteem and optimism in Italian high school students.

The first hypothesis of the second study was confirmed as the participants with self-fulfilling profiles demonstrated higher self-esteem than the participants with the other profiles. These results are in line with those of studies carried out with Swedish participants (Archer et al., 2008) and with Swedish as well as Indonesian participants (Adrianson et al., 2013). Individuals with more positive emotions with regard to pleasure attainment and fewer negative emotions with regard to pain avoidance generally have higher self-esteem. These self-fulfilling individuals, who simultaneously have a high PA and a low NA, seem to have a more positive overall assessment of their self-worth (Archer et al., 2008; Adrianson et al., 2013).

The second hypothesis of the second study was confirmed as the participants with self-fulfilling profiles demonstrated higher optimism than the participants with the other affective profiles. These results are in line those of studies carried out with Swedish participants (Archer et al., 2008) and with Swedish as well as Indonesian participants (Adrianson et al., 2013). Individuals who experience more positive emotions in terms of both high PA and low NA are generally more optimistic thus indicating that self-fulfilling individuals tend to judge the course of events favorably and are positive about facing the problems and negative events of life (Archer et al., 2008; Adrianson et al., 2013).

Despite these results, it is important to point out the research limitations inherent in the exclusive use of a group of Italian students at a high school in Tuscany who are not necessarily representative of Italian high school students in general just as Tuscany is not necessarily representative of other geographical areas in Italy. Future studies should, therefore, consider involving participants who are more representative of the Italian reality, for example high school students of different ages and from other regions in Italy. Future studies could also involve other populations such as university students and adults. The present study could also be replicated in international contexts to see whether the affective profiles model yields similar results with, for example, multiethnic samples of adolescents (Villodas et al., 2011).

Notwithstanding these limitations, the present study showed that also in the Italian context individuals with self-fulfilling profiles, that is, individuals who frequently experience positive emotions and infrequently experience negative emotions, experience greater life satisfaction, psychological well-being, self-esteem, and optimism. These results are consistent with those across different international contexts suggesting that the affective profiles model holds true in different contexts (Di Fabio, 2014a; Di Fabio et al., in press; Di Fabio and Saklofske, 2014b). The results further suggest that self-fulfilling profiles can be promoted through interventions based on specific training (Ricciardi et al., 2014). This prospect is particularly promising for adolescents who face important career decisions and transitions in their lives and who are at a critical and challenging stage regarding their positive self and relational management (Di Fabio, 2015).

A positive psychology approach (Seligman and Csikszentmihalyi, 2000; Seligman, 2002) should focus on the promotion of individual resources (Di Fabio, 2006, 2015; Di Fabio and Bernaud, 2008; Di Fabio and Kenny, 2012a,b, 2015; Di Fabio and Maree, 2012; Di Fabio et al., 2012; Di Fabio and Saklofske, 2014a) taking into account the affective profiles model, which can help individuals derive greater life satisfaction and psychological well-being on the one hand and a more positive perspective on themselves and on their lives on the other hand.

## Conflict of Interest Statement

The authors declare that the research was conducted in the absence of any commercial or financial relationships that could be construed as a potential conflict of interest.
